# Altered muscle coordination when pedaling with independent cranks

**DOI:** 10.3389/fphys.2013.00232

**Published:** 2013-08-28

**Authors:** François Hug, Florian Boumier, Sylvain Dorel

**Affiliations:** ^1^Laboratory “Motricité, Interactions, Performance” (EA 4334), UFR STAPS, University of NantesNantes, France; ^2^NHMRC Centre of Clinical Research Excellence in Spinal Pain, Injury and Health, School of Health and Rehabilitation Sciences, The University of QueenslandBrisbane, QLD, Australia

**Keywords:** electromyography, cycling, training, pedaling, muscle contraction

## Abstract

Pedaling with independent cranks ensures each leg cycles independently of the other, and thus eliminates the contribution of the contralateral leg during the upstroke phase. Consequently the subject is required to actively pull-up the pedal to complete the cycle. The present study aimed to determine the acute effect of the use of independent cranks on muscle coordination during a submaximal pedaling exercise. Ten healthy males were asked to perform submaximal pedaling exercises at 100 Watts with normal fixed cranks (control condition) or independent cranks. Both 2-D pedal forces and electromyographic (EMG) SIGNALS of 10 lower limb muscles were recorded. When the mean EMG activity across the cycle was considered, the use of independent cranks significantly increased the activity level compared to control for Tibialis anterior (TA) (*P* = 0.0017; +336 ± 302%), Gastrocnemius medialis (GM) (*P* = 0.0005; +47 ± 25%), Rectus femoris (RF) (*P* = 0.005; +123 ± 153%), Biceps femoris (BF)—long head (*P* = 0.0001; +162 ± 97%), Semimembranosus (SM) (*P* = 0.0001; +304 ± 192%), and Tensor fascia latae (*P* = 0.0001; +586 ± 262%). The analysis of the four pedaling sectors revealed that the increased activity of hip and knee flexors mainly occurred during the top dead center and the upstroke phase. In addition, a high inter-individual variability was found in the way the participants adapted to pedaling with independent cranks. The present results showed that the enforced pull-up action required when using independent cranks was achieved by increasing the activation of hip and knee flexors. Further studies are needed to determine whether training with independent cranks has the potential to induce long-term changes in muscle coordination, and, if so, whether these changes are beneficial for cycling performance.

## Introduction

Cycling performance depends on many physiological and biomechanical factors. A proper technique is generally believed to improve the gross efficiency (Leirdal and Ettema, 2011). As 1-h of pedaling corresponds to about 4800 crank revolutions, even a small increase in pedaling effectiveness would induce significant gains in performance.

Taking advantage of the development of instrumented pedals, some studies have analyzed pedal force profiles in untrained participants (Dorel et al., 2010) and trained cyclists (Korff et al., 2007; Dorel et al., 2009). As the two cranks are connected to each other, the force produced during the downstroke phase automatically lifts the contralateral pedal. Because of the effect of gravity on the lower limb, negative torque is classically measured during the upstroke phase (also called “recovery” phase), when exercise intensity is submaximal (Coyle et al., 1991; Dorel et al., 2009). As it has been shown that an active pull-up on the pedal results in a greater mechanical effectiveness (Korff et al., 2007; Mornieux et al., 2008), there is a growing interest in the cycling community for training this pull-up action.

Independent cranks (e.g., PowerCranks, PowerCranks Inc, CA, USA) have been designed to improve cycling performance. As the two cranks are independent of each other, the contribution of the contralateral leg during the upstroke phase is eliminated, forcing the cyclist to actively pull-up the pedal. This training system is thought to increase activation of both hip and knee flexors, and is used as a training intervention for strengthening leg muscles and/or for improving pedaling technique (i.e., mechanical effectiveness). However, as far as we know only two studies have explored the effect of cycling with independent cranks on muscle coordination (Fernandez-Pena et al., 2009; Burns et al., 2012). These studies focused on the long-term effect of using this system on the myoelectrical activity of three to four lower limb muscles. Consequently, information about the acute effects on muscle activity is unknown. Further, no previous study has considered the activity of the hip flexors, which is thought to be increased considerably using this training technique.

A complete understanding of the acute effect of using independent cranks on muscle coordination is crucial for coaches/physiotherapists to be able to formulate appropriate specific training/rehabilitation programs. More specifically, it is important to determine which muscles are affected by the use of independent cranks. Therefore, the present study aimed to determine the acute effect of the use of independent cranks on muscle coordination during a submaximal pedaling exercise. Muscle coordination was assessed in 10 lower limb muscles using electromyography (EMG), in terms of both the EMG activation profiles and EMG amplitude. In addition, an instrumented pedal was used to measure the 2D forces at the shoe-pedal interface. We hypothesized that the use of independent cranks would increase the activity level of muscles involved in the upstroke phase during normal pedaling (Tensor fascia latae, TF). Further, although hamstrings are mainly activated during the downstroke phase (as hip extensors) during normal pedaling (Hug and Dorel, 2009), they are also able to produce positive torque around the bottom dead center and during the upstroke phase (as knee flexors) because of their biarticular function. Consequently, we also hypothesized that the use of independent cranks would increase the activity level of hamstrings during the upstroke phase (a phase of the pedaling cycle where they normally have no or little activation).

## Materials and methods

### Participants

Ten healthy males, with no history of cycling training, participated in this experiment (age: 24.6 ± 4.1 years, height: 178 ± 4 cm, body mass: 73.2 ± 7.8 kg). The participants were informed of the possible risk and discomfort associated with the experimental procedures prior to giving their written consent to participate. The local ethics committee (University of Nantes) approved the study, and all the procedures conformed to the Declaration of Helsinki (last modified in 2004).

### Protocol

Participants exercised on a magnetic braked cycle ergometer (Indoortrainer®, SRM, Germany) equipped with adjustable dual-mode PowerCranks (length = 170 mm; PowerCranks Inc, CA, USA). This crank system can be used either as normal fixed cranks or as independent cranks. After a 10-min sub-maximal pedaling exercise with normal fixed cranks (referred to as “Control condition”), the participants were asked to pedal with independent cranks during 1-min bouts. As pedaling with independent cranks requires a short-term learning (familiarization) period, this exercise was repeated 8 times (1-min of passive recovery between each) and only the 8th bout was analyzed (referred to as “independent cranks” condition). All cycling bouts were performed at 100 Watts and at a constant pedaling rate (70 rpm). The short duration (1-min) and the relatively low intensity of exercise were chosen to avoid occurrence of neuromuscular fatigue that was observed during pilot experiments performed at a higher workload and during longer pedaling exercises. When cycling with independent cranks, participants were instructed to pedal as naturally as possible keeping the two cranks at 180° (anti-phased). Surface EMG of 10 lower limb muscles and pedal forces were recorded continuously during each condition.

### Material and data collection

#### Pedal force measurements

The cycle ergometer was equipped with instrumented pedals (with the LOOK Keo clipless platform) specifically designed for measuring pedal loads (VélUS group, Department of Mechanical Engineering, Sherbrooke University, Canada) as previously described elsewhere (Dorel et al., 2010). Briefly, the sagittal plane components of the total reaction force (F_tot_) applied at the shoe/pedal interface were measured using a series of eight strain gauges located within each pedal. F_tot_ was calculated from the measured Cartesian components F_T_ and F_N_, corresponding respectively to the horizontal forward and vertical upward forces on the pedal. An optical encoder with a resolution of 0.4° mounted on the pedal measured pedal angle with respect to the left crank orientation. Zero adjustments for both components of force and pedal angle were carried out before each session. The left crank angle was measured using an incremental encoder. All the mechanical signals were digitized at a sampling rate of 1 kHz (ME6000, Mega Electronics Ltd., Kuopio, Finland).

#### Surface electromyography

Surface EMG was recorded on 10 lower limb muscles of the left leg: tibialis anterior (TA), soleus (SOL), gastrocnemius medialis (GM) and lateralis (GL), vastus lateralis (VL), rectus femoris (RF), vastus medialis (VM), long head of biceps femoris (BF), semimembranosus (SM), and tensor fasciae latae (TF). For each muscle, a pair of self-adhesive Ag/AgCl electrodes (Blue sensor N, Ambu, Denmark) was attached to the skin with an inter-electrode distance of 20 mm (center-to-center). The electrodes were located according to the recommendations of SENIAM (Surface EMG for Non-Invasive Assessment of Muscles) (Hermens et al., 2000). Skin was shaved and cleaned with alcohol and ether to minimize impedance before applying the electrodes. The wires connected to the electrodes were well secured with adhesive tape to avoid movement-induced artifacts. Raw EMG signals were amplified close to the electrodes (gain 375, bandwidth 8–500 Hz) and digitized at a sampling rate of 1 kHz (ME6000, Mega Electronics Ltd., Kuopio, Finland).

### Data analysis

All data were processed using Matlab (The MathWorks, USA) and Origin (OriginLab Corporation, USA).

#### Mechanical data

Mechanical signals were smoothed by a 10 Hz low-pass filter and resampled (one value per degree). On the basis of normal and horizontal components and pedal angle, the total resultant force (F_tot_) was calculated by trigonometry and resolved to determine the effective component (F_eff_, i.e., the propulsive component applied perpendicularly to the crank arm). The instantaneous index of mechanical effectiveness (IE) was determined as the ratio of F_eff_ to F_tot_ at each point of the pedaling cycle. The overall index of effectiveness on the complete crank cycle (IE_cycle_) was determined as the ratio of the linear impulse of F_eff_ to the linear integral of F_tot_ (Sanderson and Black, 2003; Dorel et al., 2009). A set of 30 consecutive cycles was extracted from a period of constant power output (between the 9th and 10th minute for the Control condition and after the first 10 s for the independent cranks condition) and averaged to obtain representative profiles of F_eff_, F_tot_, and IE.

#### EMG data

EMG signals were filtered with a bandpass filter (4th order Butterworth) between 20 and 450 Hz. Linear envelopes for each muscle were obtained by low-pass filtering the fully rectified EMG signals with an 9 Hz low-pass filter (zero lag). The same 30 consecutive cycles used for mechanical data were extracted and averaged to obtain a representative pattern for each muscle. The mean value over this pattern was considered as the mean EMG activity level. Mean values corresponding to four functional angular sectors of the pedaling cycle were also calculated (Hug et al., 2008): sector 1 represented 330–30° (top dead center), sector 2 30–150° (downstroke phase), sector 3 150–210° (bottom dead center) and sector 4 210–330° (upstroke phase) (with 0° corresponding to the highest position of the pedal). To compare the shape of EMG patterns, ensemble-averaged envelopes were normalized by the average of their peak from all cycles (Hug et al., 2010).

### Statistics

Stata 12 software (StataCorp LP, Texas, USA) was used for statistical analysis of the data. The effect of pedaling with independent cranks on IE was tested using a paired-t-test (significance level: *P*-value ≤ 0.05). As our analysis concerned changes in activity between two conditions and did not involve comparison between muscles, no normalization of EMG was performed. Separate paired-t-tests were performed to test the effect of the use of independent cranks on the mean EMG activity level calculated over whole the cycle or over each of the 4 sectors. If the Shapiro-Wilk test of normality was significant (indicating that the data were not normally distributed) a Wilocoxon signed-rank test was performed. To account for the multiple-comparison, a Bonferroni correction was applied resulting in a significance level set at *P*-value ≤ 0.005 (i.e., 0.05/10 muscles).

## Results

### Mechanical data

**Figure 1:**
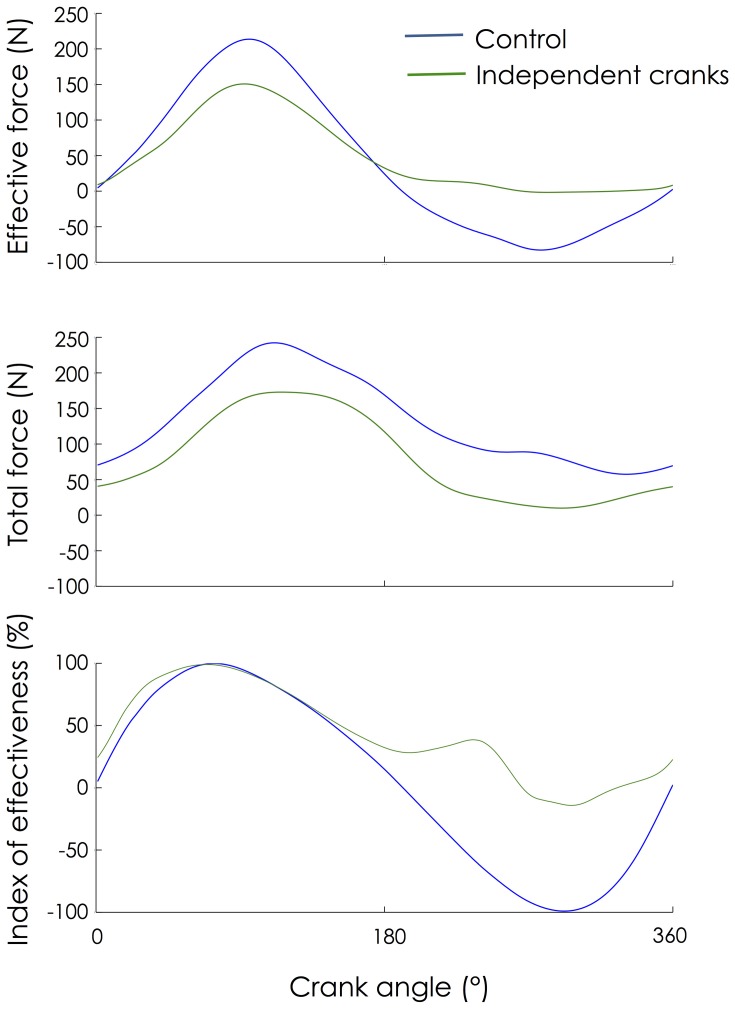
**Change in mechanical patterns**. Mechanical patterns from the left pedal were averaged across 30 consecutive pedaling cycles and expressed as a function of the left crank angle as it rotated from the highest pedal position (0°) to the lowest pedal position (180°) and back to the highest position.

As expected, the effective force was dramatically increased during the upstroke phase (becoming consistently positive) when pedaling with independent cranks compared to Control condition (Figure 1). As the same power output was maintained in each condition, this increased effective force was accompanied by a decrease in effective force during the downstroke phase. In addition, the magnitude of total force was lower in all parts of the cycle. Finally, IE increased during the upstroke phase where it became almost positive. In this way, IE_cycle_ was significantly higher (*P* < 0.001) with independent cranks (61.6 ± 6.9%) compared to control (29.1 ± 4.4%).

### EMG profile

**Figure 2:**
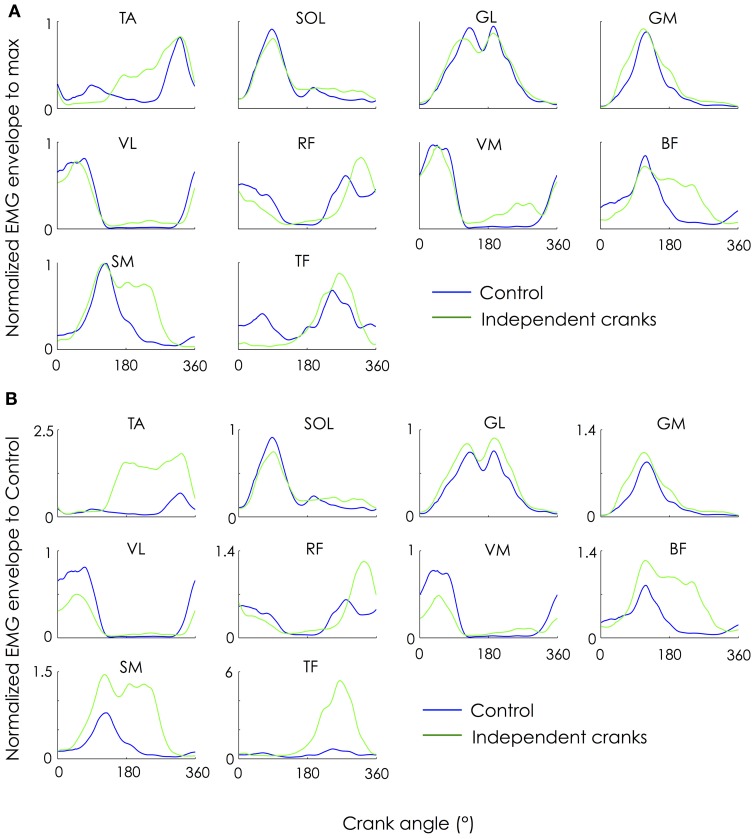
**Change in ensemble-averaged EMG patterns**. EMG patterns were averaged across 30 consecutive pedaling cycles and expressed as a function of the left crank angle as it rotated from the highest pedal position (0°) to the lowest pedal position (180°) and back to the highest position. To compare the shape of the EMG patterns, the amplitude was first normalized for each condition by the average of its peak from all cycles (panel **A**). To compare both the shape and the amplitude of the EMG patterns, the amplitude was then normalized by the average of its peak from all cycles measured during the control condition (panel **B**). TA, tibialis anterior; SOL, soleus; GL, gastrocnemius lateralis; GM, gastrocnemius medialis; VL, vastus lateralis; RF, rectus femoris; VM, vastus medialis; BF, long head of biceps femoris; SM, semimembranosus; TF, tensor fascia latae.

For each condition, the normalized EMG patterns for the 10 muscles investigated are shown in Figure 2. Overall, EMG patterns of the Control condition were similar to those already reported in the literature (for a review, see Hug and Dorel, 2009). When the shape of the EMG patterns is considered (patterns normalized for each condition to the average of the peaks amplitude; Figure 2A), it appears that some muscles were more affected by the use of independent cranks (e.g., TA, BF, SM, and TF). Note that TA, BF and SM were active during a longer period of the cycle when pedaling with independent cranks compared to Control (Figure 2A). When patterns normalized to the average of the peaks amplitude of the Control condition are considered (Figure 2B), it clearly appears that the magnitude of activation of some muscles was greatly altered by the use of independent cranks, as quantified below.

### EMG amplitude

**Figure 3:**
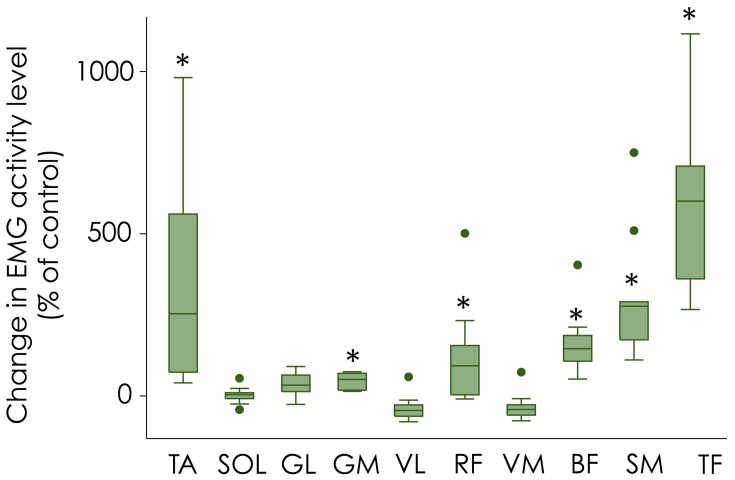
**Change in muscle activity level (mean value over the crank cycle)**. Changes in EMG amplitude normalized to the control condition are shown with box plots for each muscle. Error bars denote the 95% confidence interval; box denotes the 25–75 percentile with the median. Dots corresponds to the outliers for muscle abbreviations see Figure 2. ^*^Significantly different from control.

Figure 3 depicts the changes in EMG amplitude (mean value over the crank cycle) normalized to the control condition. The statistical analysis performed on raw data showed that the use of independent cranks significantly increased muscle activity level compared to control for TA (*P* = 0.0017; +336 ± 302%), GM (*P* = 0.0005; +47 ± 25%), RF (*P* = 0.005; +123 ± 153%), BF (*P* = 0.0001; +162 ± 97%), SM (*P* = 0.0001; +304 ± 192%), and TF (*P* = 0.0001; +586 ± 262%). According to the Bonferroni correction, no significant effect was found for the other muscles (SOL—*P* = 0.76; GL—*P* = 0.029; VL—*P* = 0.012; VM—*P* = 0.0312).

**Figure 4:**
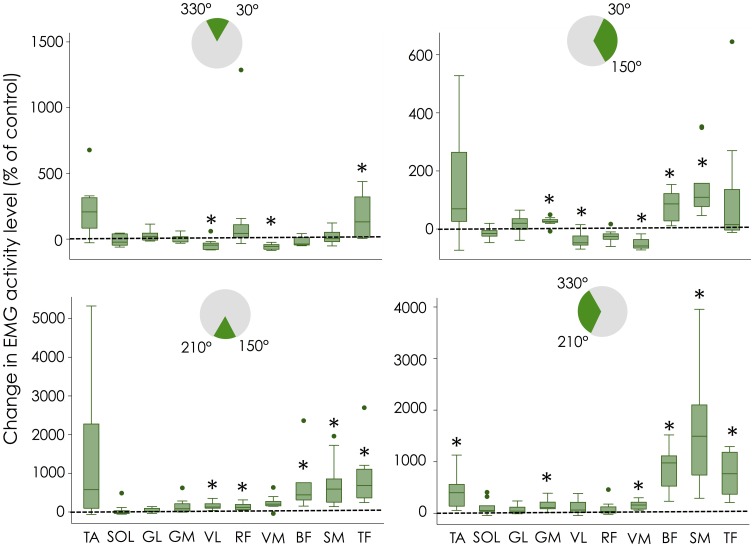
**Change in muscle activity level for each pedaling sector**. Changes in EMG amplitude normalized to the control condition are shown with box plots for each muscle. Error bars denote the 95% confidence interval; box denotes the 25–75 percentile with the median. Dots corresponds to the outliers for muscle abbreviations see Figure 2. Note that the y-scale is different for each graph. ^*^Significantly different from control.

When each pedaling sector was analyzed individually (Figure 4), a significant decrease in EMG amplitude was found for VL and VM (*P* = 0.005; −54 ± 22% and −56 ± 22%, respectively) compared to Control during sector #1 (330–30°; top dead center) while TF activity was significantly increased (*P* = 0.005; +171 ± 165%). During sector #2 (30–150°; downstroke phase) a significant decrease in both VL (*P* = 0.0003; −44 ± 16%) and VM (*P* = 0.0003; −50 ± 19%) activity was found while activity of GM (*P* = 0.0013; +25 ± 15%), BF (*P* = 0.0013; +79 ± 49%) and SM (*P* < 0.0001; +148 ± 111%) increased. During sector #3 (150–210°; bottom dead center) a significant increase in activity was found for VL (*P* < 0.0001; +159 ± 92%), RF (*P* = 0.005; +135 ± 88%), BF (*P* < 0.0001; +645 ± 645%), SM (*P* = 0.005; +766 ± 618%), and TF (*P* = 0.005; +862 ± 723%). It is important to note that the increase in VL activity (although significant) is unlikely to have important functional implications, as this knee extensor muscle has limited activation during this sector. Finally, during sector #4 (210–330°; upstroke phase), 6 muscles exhibited a significant increase in activation, i.e., TA (*P* = 0.0008; +423 ± 328%), GM (*P* = 0.0013; +178 ± 145%), VM (*P* = 0.0001; +162 ± 81%), BF (*P* = 0.005; +919 ± 411%), SM (*P* = 0.0004; +1600 ± 1084%), and TF (*P* = 0.005; +767 ± 389%). Again, the significant increase in VM activity should be interpreted with caution, as this muscle acts as an antagonist muscle during this sector.

## Discussion

The aim of the present study was to determine the effect of the use of independent cranks on muscle coordination during a submaximal pedaling exercise. When changes in both the shape and the amplitude of the EMG patterns are considered, TA, Hamstrings (BF and SM), RF and TF were found to be the muscles most affected by the use of independent cranks.

### Functional significance

In accordance with our hypothesis, the enforced pull-up action required by using independent cranks was achieved by increasing the activation of hip and knee flexors. More precisely, the use of independent cranks dramatically increased the muscle activity levels of TA, SM, TF, and, to a lesser extent, BF and RF (Figures 3, 4). This overall increase in lower limb muscle activity is consistent with the observation that actively pulling-up on the pedals (while normal cranks are used) induces a decrease in gross efficiency (Korff et al., 2007). This increased activity was accompanied by a decrease in activity of monoarticular knee extensors (VL and VM) during sector #2 (30–150°; downstroke phase) that explains the decrease in effective force during this phase (Figure 1). As the same power output was maintained during both conditions (i.e., Control and independent cranks), the reduced work produced during the downstroke phase when cycling with independent cranks can be logically explained by the increased work produced during the upstroke phase. Interestingly, these adaptations have been shown to be transferred to a normal pedaling condition after a training period with independent cranks (Bohm et al., 2008).

In addition to these alterations of muscle activity level, cycling with independent cranks altered the functional role of some muscles. Inspection of both the muscle activation profiles (Figure 2) and activation amplitude per sector (Figure 4) confirmed our hypothesis that hamstrings which are normally active at the end of the downstroke phase, were also active through the entire upstroke phase when cycling with independent cranks. This is in contrast to a study from Mornieux et al. (2010), who reported no change in the timing of BF activity when the subjects were instructed to actively pull-up on the pedal while they used normal cranks. Consequently, instructing the participants to actively pull-up on the pedal is unlikely to reproduce the effects induced by the use of independent cranks.

While TA (dorsiflexor) was mainly activated around the top dead center (highest position of the pedal) during the control condition, it exhibited an increase in activation during whole of the upstroke phase during the independent cranks condition. This adaptation likely occurred in order to transmit the additional knee/hip flexion force to the pedal. Finally, among the triceps surae (plantarflexors), only GM was affected by the use of independent cranks. This further confirms that GM and GL, although belonging to the same muscle group, may receive a different neural activation (Ahn et al., 2011).

**Figure 5:**
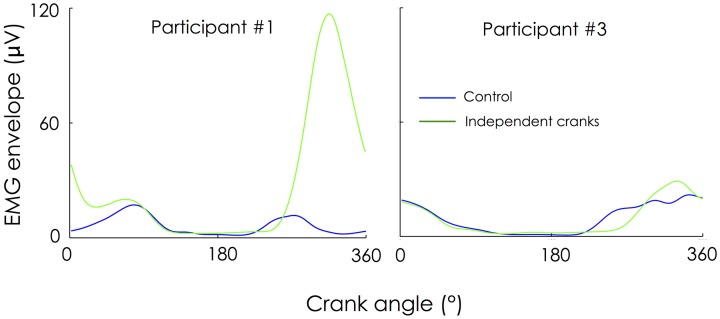
**Example of two different adaptations for the rectus femoris**. EMG envelopes are not normalized (expressed in microvolts) and thus contain information on both muscle activity level and distribution of this activity within the cycle. While participant #1 increased RF activity level by about 500% during the “independent cranks” condition, RF recruitment was not altered in participant #3.

As revealed by the large 95% confidence intervals on Figures 3, 4, there was a high inter-individual variability in the way the participants adapted to these new mechanical constraints. This is particularly clear for TA, RF, and TF. For instance, 3 out of 10 participants did not exhibit any change in RF activity while the others exhibited an increase in activity ranging from +40 to +500%. Figure 5 depicts an example of two different behaviors observed for RF. While participant #1 demonstrated an increased RF activity level by about 500% during the independent cranks condition, RF recruitment was not altered in participant #3. Such an inter-individual variability in muscle coordination has already been reported during normal pedaling in trained cyclists, especially for biarticular muscles (Hug et al., 2008, 2010). The present results further show that variability also exists in the way that participants adapt to mechanical constraints (independent cranks here).

### Methodological considerations

The limitations of the present study are inherent to the use of surface EMG. First, it is possible that crosstalk occurred, especially for small muscles such as TF. As crosstalk may be reduced by a proper localization of the surface electrodes on the muscle (Hug, 2011), we followed the SENIAM recommendations (Hermens et al., 2000). As TF EMG activity was mainly localized during the upstroke phase, if crosstalk occurred, it occurred with another hip flexor muscle (e.g., psoas major). Taken together with the fact that changes in EMG amplitude were large (e.g., +586 ± 262% for TF), we are confident that crosstalk does not preclude any interpretation/conclusion from the present data.

As the aim of the present study was to quantify change in muscle coordination from a control condition, no normalization of EMG data was required. Consequently, information regarding the degree of muscle activity (which requires to refer to the maximal EMG activity level) is missing. Although this information would have been useful to better quantify the muscular demand associated to the use of independent cranks, determination of the maximal EMG activity level is not straightforward (Dorel et al., 2012). To date there is no agreement on the best normalization procedure to be used (Burden, 2010; Hug, 2011).

### Practical implications and perspectives

The present data hold useful information for the formulation of appropriate cycling training programs and rehabilitation protocols. The magnitude of change in muscle activity level observed in dorsiflexors, knee flexors and hip flexors provides evidence that the use of these specific cranks acts as a specific strength-training program.

It is less clear whether training with independent cranks has the potential to alter durable muscle coordination when normal cranks are used. Results from Fernandez-Pena et al. (2009) suggested small alterations in the coordination patterns that are easily lost if the independent crank training is not continued. However, in this latter study, the authors did not measure SM and TF muscles that were affected most in the present study. Further studies are thus needed to determine whether training with independent cranks has the potential to alter muscle coordination, and, if so, whether these changes are beneficial for cycling performance.

Interestingly, the present study showed variability in the way the participants adapted to new mechanical constraints. Hence, it is possible that the gain in pedaling technique (if any) related to the long-term use of independent cranks differs between individuals as well. This might explain the failure of previous studies to show any systematic effect of training with independent cranks either in muscle coordination, or gross efficiency (Burns et al., 2012). In other words, long-term benefits might depend on the coordination strategy adopted when cycling with uncoupled cranks, thus raising questions about the relevance of the use of a learning period prior using independent cranks within a training program.

## Grants

This study was funded by the French Ministry of Sport (contract 10-R-019) and the Region Pays de la Loire (ANOPACy project).

### Conflict of interest statement

The authors declare that the research was conducted in the absence of any commercial or financial relationships that could be construed as a potential conflict of interest.
